# 

**Published:** 1896-11-21

**Authors:** 


					/
THE HOSPITAL. ABSOLUTELY PURE, therefore BEST, Nov. 21st, 1896.
CADBURY'S CADBURY'S
OOOOA
"The standard of highest purity at present \=* ?=/ w Mr> .T itit tvb ttovt>
attainable.'? Lancet. c NO ALKALIKS USKD,
jn
OOOOA
aJltJffiS1 _ __
Lasco nr Wes tern
QRW1CH HOSPLTAJ.
E TWOFE]
nam, 10a 6d., post
rT>??,*0<-?-ori of +V10 ftpnornl Post Office aa a NswanaDer for Monthly Parts. 6d.: nost frno. Rrt.
No/JoTvol xTl. WITH EXTRA NURSING SUPPLEMENT. nggr^wgmngw.
Saturday,
Nov. 21st, 1896.
[Registered at the wenerai rost umce as a newspaper ior Montniy raixs, oa.; posu ireef?a.
transmission in the United Kingdom and Abroad,] Ditto per Annum, 8s. 6d., post fre*t

				

